# A Histopathological Study of Endometrial Biopsy Samples in Abnormal Uterine Bleeding

**DOI:** 10.7759/cureus.31264

**Published:** 2022-11-08

**Authors:** Ashi Vijayaraghavan, Chaithra Jadhav, Bharathi Pradeep, Hima Bindu, Senthil Kumaran

**Affiliations:** 1 Pathology, Sri Lakshmi Narayana Institute of Medical Sciences, Puducherry, IND; 2 Pathology, Indira Gandhi Medical College and Research Institute, Puducherry, IND; 3 Obstetrics and Gynaecology, Indira Gandhi Medical College and Research Institute, Puducherry, IND

**Keywords:** atypical endometrial hyperplasia, endometrial hyperplasia, endometrial carcinoma, endometrial sampling, abnormal uterine bleeding

## Abstract

Background: Abnormal uterine bleeding (AUB) is the most common health issue in women, defined as any bleeding pattern that differs in frequency, duration, and amount. Endometrial sampling and its histopathological examination is the first-line test in patients presenting with AUB. The aim of this study was to analyze the histopathological patterns of endometrium in women with AUB and to find the predominant histopathologic pattern in the different age groups of women with AUB.

Methods: The study was conducted at the Indira Gandhi Medical College and Research Institute, Puducherry, from January 2019 to December 2020. Endometrial biopsies of patients with AUB, in whom gestational causes were ruled out, were included in this study.

Results: Out of the 160 cases analyzed, the maximum number of biopsies were from the age group of 41-50 years; the majority of patients presented with complaints of menorrhagia. The bleeding pattern was significantly associated with age groups (p=0.00). Of 160 cases, 104 cases were related to functional causes. The association of functional and organic causes with age group was not significant (p=0.67 and p=0.99, respectively). The most common histological pattern was the normal cyclical pattern showing proliferative (56) and secretory phase (30) in 86 cases. Of 42 cases of endometrial hyperplasia, 9 cases had atypical hyperplasia. The endometrial polyp was the other common organic lesion observed. Only two cases of endometrial carcinoma were reported during the present study period.

Conclusion: Although a regular cyclical pattern is observed commonly, endometrial sampling should be considered in the peri- and post-menopausal age groups wherein the incidence of endometrial hyperplasia and endometrial carcinoma is more common.

## Introduction

Abnormal uterine bleeding (AUB) is the most common health issue seen in women of all age groups. AUB is defined as any bleeding pattern that differs in frequency, duration, and amount from a pattern observed during normal menstrual cycles or menopause. Bleeding is said to be abnormal when the pattern is irregular, of abnormal duration (seven days), or of abnormal amount (>80 ml/menses). AUB is the major gynecological problem responsible for as many as one-third of all outpatient gynecological visits. It has varied presentations like heavy menstrual bleeding (HMB), frequent cycles, irregular cycles, post-coital bleeding, or post-menopausal bleeding (PMB). It affects women of every age group from adolescence to menopause. It reflects the underlying pathology as simple as hormonal imbalance or carcinoma requiring aggressive treatment. AUB has a significant effect on the quality of life of women [1]. AUB is due to several factors deranging homeostasis like hormonal imbalances, infections, structural lesions, and malignancy. Based on these possible underlying etiologies, the International Federation of Gynaecology and Obstetrics (FIGO) in 2011 devised a classification named PALM-COEIN for the etiology of AUB. PALM accounts for structural features like polyps, adenomyosis, leiomyoma, and malignancy. COEIN addresses non-structural causes like coagulation defects, ovulatory dysfunction, endometrial causes, iatrogenic causes, and non-classified ones [2]. Endometrial biopsy is used as a diagnostic aid in AUB. It is done as a first-line test in women >45 years of age presenting with AUB. Endometrial biopsy is also done in patients <45 years of age with a history of unopposed estrogen exposure, failed medical management, and persistent AUB [3]. The prime idea is to rule out the precursor lesions like hyperplasia and aggressive endometrial carcinoma [2]. The present study was done to determine the histopathological spectrum of endometrium in women presenting with abnormal uterine bleeding.

## Materials and methods

This was a hospital-based retrospective observational study conducted in the Department of Pathology in collaboration with the Department of Obstetrics and Gynaecology, Indira Gandhi Medical College and Research Institute, Puducherry, over a period of two years from January 2019 to December 2020 with approval (No.32/IEC-32/IGMC&RI/PP-1/2021) from the Institute Ethics Committee (Human Studies).

The study included endometrial samples of 160 patients who had been advised for endometrial sampling for non-gestational causes. Endometrial curettage done in the case of AUB due to gestational causes like incomplete abortion, missed abortion and retained products of conception was excluded from the study. All endometrial biopsies from patients with AUB not due to gestational causes were included in the study. The relevant clinical details like age, presenting complaints, and menstrual details including last menstrual period, periodicity, and regularity were collected from the case records of patients. Hematoxylin and eosin stained slides were examined thoroughly and the findings were recorded. Ziehl-Neelsen stained sections were also examined as per requirement.

The data collected regarding age, relevant clinical findings, and histopathological diagnosis were statistically analyzed using IBM SPSS Statistics, version 21 (IBM Corp., Armonk, NY). Variables were summarized using frequency and percentage. The chi-square test was performed to analyze the categorical variables. Fisher's exact test was utilized for checking the association between categorical variables. A p value below 0.05 was considered statistically significant.

## Results

In this study, a total of 160 endometrial biopsies done in patients with AUB sent for histopathological examination findings were analyzed. Based on the age of the patients who underwent endometrial sampling, data was categorized into three groups: reproductive, perimenopausal and post-menopausal. Perimenopausal category had 56.3% of cases that was the maximum among all categories. It was observed that 28.1% of the patients with AUB fell in the reproductive age group. The post-menopausal category had 15.6% of patients, which was the least.

It was observed that menorrhagia was the most common bleeding pattern accounting for 71.25% cases. Hypomenorrhea was the least common bleeding pattern that constituted only two patients (1.2%). Metrorrhagia and PMB patterns were observed in 15% and 12.5% of the study population, respectively.

Age-specific analyses clearly revealed that menorrhagia is the most common complaint seen in the perimenopausal age group and reproductive age group and was significantly associated (p=0.00). The second most common complaint seen in the reproductive and perimenopausal age groups was metrorrhagia. A total of 20 cases presented with complaints of post-menopausal bleeding (Table 1).

**Table 1:** Age-wise distribution of the bleeding pattern in patients with abnormal uterine bleeding

S. no.	Bleeding pattern	Age 18-40	Age 41-50	Age >50	Total (%)
1	Menorrhagia	27	78	9	114 (71.25%)
2	Metrorrhagia	16	7	1	24(15%)
3	Hypomenorrhea	2	0	0	2 (1.25%)
4	Post-menopausal bleeding	0	5	15	20 (12.5%)
5	Total	45	90	25	100

Among the endometrial sampling of 160 AUB patients, the commonest histopathological pattern observed among all age groups was proliferative phase endometrium (35%). The second most common pattern, endometrial hyperplasia without atypia was seen in 33 patients (20.6%); atypical endometrial hyperplasia was seen in only nine patients (5.7%). The third was secretory phase endometrium seen in 30 patients (18.8%). The next histopathological pattern was disordered proliferative endometrium that accounted for 7% cases. The other various patterns observed in the descending order of frequency are endometrial polyp (4%), menstrual endometrium (3.1%), chronic nonspecific endometritis (1.9%), endometrial carcinoma (1.2%) and granulomatous endometritis, Arias-Stella reaction, and atrophic endometrium contributing 0.6% each.

Chronic nonspecific endometritis, granulomatous endometritis and Arias-Stella reaction were observed only in the reproductive age group. Endometrial polyp and atypical endometrial hyperplasia were noted among all the age groups. Among the cases in the perimenopausal and menopausal age groups, one case each was reported as endometrial carcinoma (Table 2).

**Table 2:** Agewise distribution of AUB due to functional and organic causes

	Histopathological pattern	Age group			Total	%
		18-40	41-50	>50		
Functional cause	Proliferative phase endometrium	14 (48.2%)	34 (54.8%)	8 (61.5%)	56	53.85
	Secretory phase endometrium	7 (24.1%)	20 (32.2%)	3 (23%)	30	28.84
	Disordered proliferative endometrium	7 (24.1%)	4 (6.4%)	1 (7.6%)	12	11.53
	Menstrual endometrium	1 (3.4%)	4 (6.4%)	0	5	4.81
	Atrophic endometrium	0	0	1 (7.6%)	1	0.97
	Total	29	62	13	104	100
Organic cause	Chronic nonspecific endometritis	1 (6.2%)	2 (7.1%)	0	3	5.36
	Granulomatous endometritis	1 (6.2%)	0	0	1	1.79
	Arias-Stella reaction	1 (6.2%)	0	0	1	1.79
	Endometrial polyp	3 (18.7%)	2 (7.1%)	2 (16.6%)	7	12.50
	Endometrial hyperplasia without atypia	7 (43.7%)	20 (71.4%)	6 (50%)	33	58.92
	Atypical endometrial hyperplasia	3 (18.7%)	3 (10.7%)	3 (25%)	9	16.07
	Endometrial carcinoma	0	1 (3.5%)	1 (8.4%)	2	3.57
	Total	16	28	12	56	100

The functional cause of uterine bleeding was considerably higher (65%) when compared to organic lesions (35%). In the reproductive and perimenopausal ages, the cause of AUB was more functional and less because of organic lesions, whereas in post-menopausal age, both functional and organic causes were responsible for AUB. The functional as well as the organic cause of AUB was not significantly associated with age group (p=0.67 and p=0.99, respectively).

The functional causes among all age groups in the decreasing order of frequency were proliferative phase endometrium (53.85%), secretory phase endometrium (28.8%), disordered proliferative endometrium (11.53%), menstrual endometrium (4.81%) and atrophic endometrium (0.97%).

**Figure 1:**
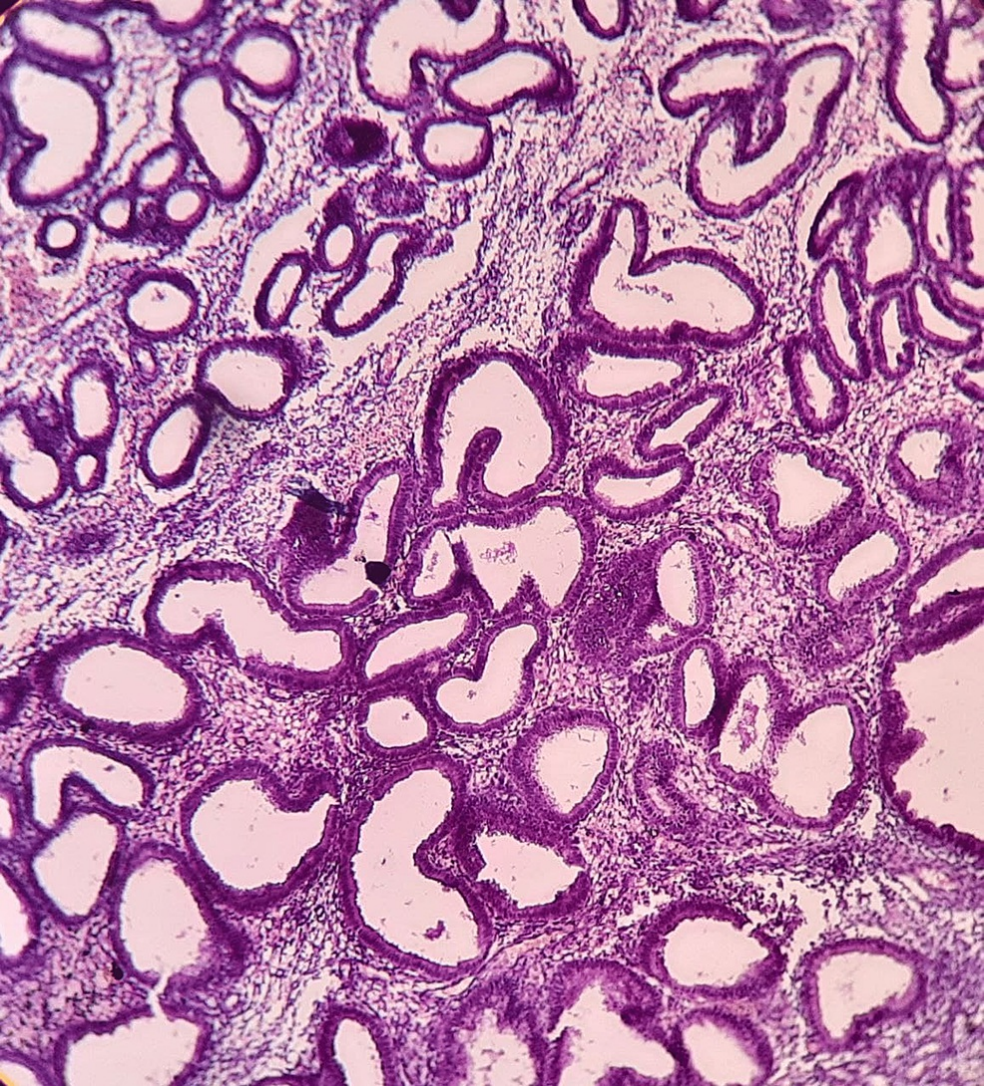
Endometrial hyperplasia without atypia (10x hematoxylin and eosin)

**Figure 2:**
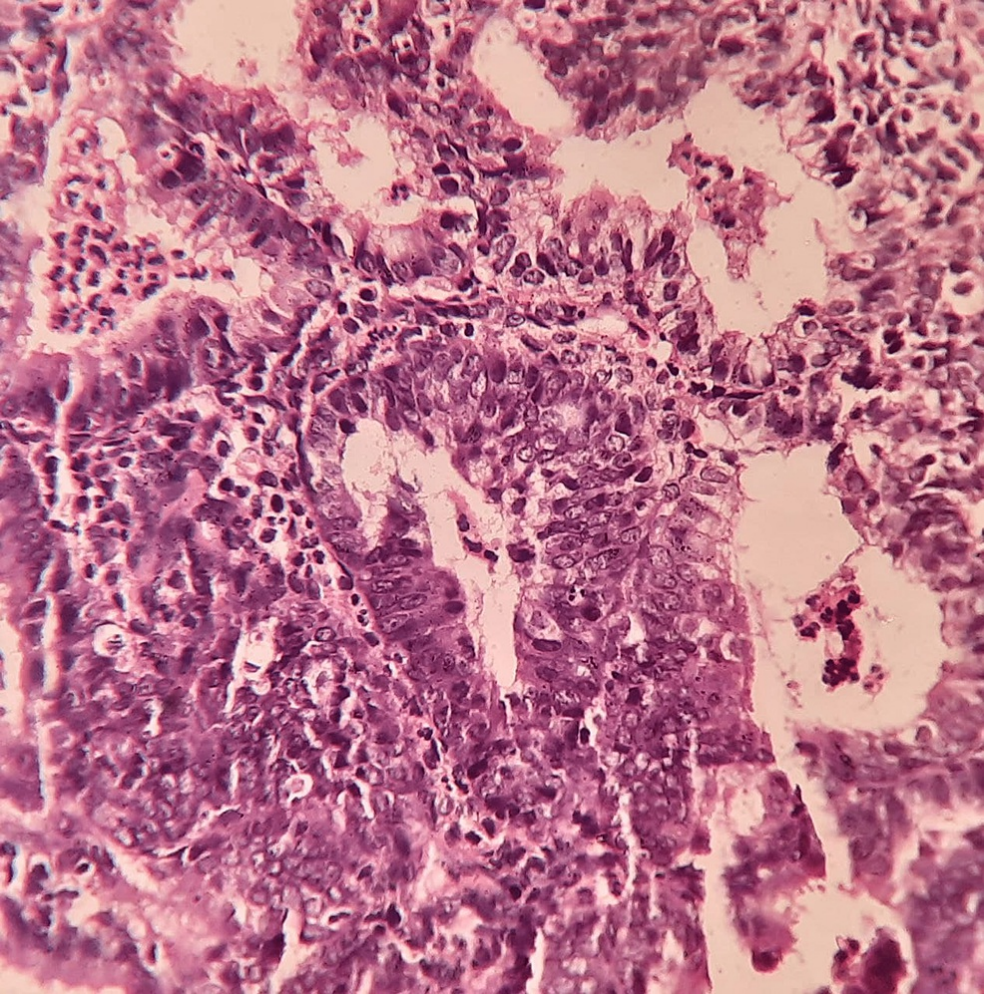
Atypical endometrial hyperplasia with the glandular endometrial lining showing mild atypia (40x hematoxylin and eosin)

**Figure 3:**
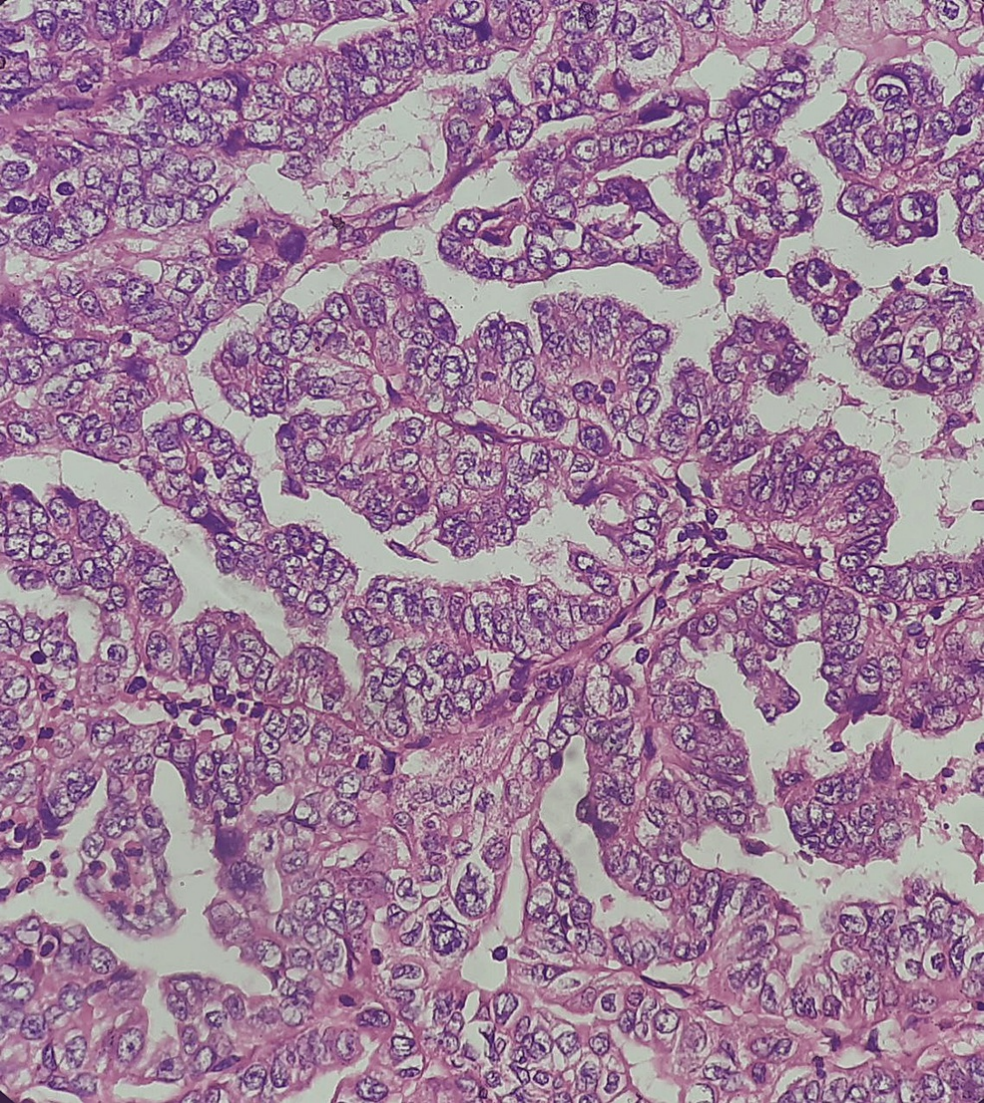
Well-differentiated endometrial carcinoma (10x hematoxylin and eosin)

AUB due to the organic cause was found in 56 patients of various age groups. Endometrial hyperplasia without atypia was the most common histological pattern among the organic causes found in 33 cases (58.9%) (Figure 1). This was followed by atypical endometrial hyperplasia (16.07%), endometrial polyp (12.5%), endometrial carcinoma (3.57%), granulomatous endometritis and Arias-Stella reaction (1.79% each) (Figures 2, 3). Among the 12 patients of the post-menopausal age group, 50% had endometrial hyperplasia without atypia, 25% had atypical endometrial hyperplasia, 16.6% had endometrial polyp and 8.4% had endometrial carcinoma.

The most common histopathological pattern observed in patients presenting with menorrhagia, metrorrhagia and post-menopausal bleeding was proliferative phase endometrium. Atypical endometrial hyperplasia and endometrial polyp presented as menorrhagia and post-menopausal bleeding in reproductive and post-menopausal age groups, respectively. Endometrial hyperplasia without atypia most commonly presented with menorrhagia in the reproductive age group. Granulomatous endometritis and Arias-Stella reaction presented with metrorrhagia. Chronic endometritis presented as metrorrhagia or menorrhagia (Table 3).

**Table 3:** Correlation between the bleeding pattern and histopathological diagnosis

Bleeding pattern	Histopathological diagnosis	No. of cases	%
Menorrhagia	Proliferative phase	39	34.21
	Secretory phase	26	22.81
	Menstrual endometrium	5	4.39
	Disordered proliferative endometrium	8	7.01
	Chronic endometritis	1	0.88
	Benign endometrial polyp	5	4.39
	Endometrial hyperplasia without atypia	23	20.18
	Atypical endometrial hyperplasia	5	4.38
	Endometrial carcinoma	2	1.75
	Total	114	100
Metrorrhagia	Proliferative phase endometrium	7	29.13
	Secretory phase endometrium	3	12.53
	Disordered proliferative endometrium	4	16.67
	Granulomatous endometrium	1	4.17
	Chronic endometrium	2	8.33
	Arias-Stella reaction	1	4.17
	Endometrial hyperplasia without atypia	5	20.83
	Atypical endometrial hyperplasia	1	4.17
	Total	24	100
Hypomenorrhea	Proliferative phase endometrium	1	50
	Secretory phase endometrium	1	50
	Total	2	100
Post-menopausal bleeding	Proliferative phase endometrium	9	45
	Atrophic endometrium	1	5
	Benign endometrial polyp	2	10
	Endometrial hyperplasia without atypia	5	25
	Atypical endometrial hyperplasia	3	15
	Total	20	100
Total number of cases		160	100

## Discussion

AUB describes bleeding per vagina that does not fall in the criteria of regular menstrual bleeding. It can be due to varied causes such as functional, organic or pharmacological agents. The etiology also varies according to the age group as well. Endometrial sampling is a safe procedure that helps to evaluate the endometrium and bring out the diagnosis, though it has the limitations of being a blind procedure.

Age distribution

A detailed analysis of the different endometrial patterns was done in this study taking into account various parameters like age, date of onset of the last menstrual period, duration of the cycle and use of any medications. The incidence of AUB was found to be the highest among the perimenopausal age group (41-50 years; 56.3%). The women in this age group were in their climacteric, and during this period, there is a decline in oestradiol levels and number of ovarian follicles resulting in anovulatory cycles. The same age group was found to be affected the most in studies by Doraiswami et al. and Sharma et al. [4,5]. The lowest incidence of 15.6% observed in the post-menopausal age group can be attributed to the early evaluation and treatment of patients, which reduced the incidence in older age groups.

Chief complaints in patients

In this study, 71.25% presented with the complaint of menorrhagia. This was the most common presenting complaint in both reproductive and perimenopausal age groups. The next frequent complaint was metrorrhagia. Menorrhagia is the commonest presentation of AUB found in studies done by Sharma et al., Sajitha et al. and Mukhopadhyay et al. [5-7].

The present study revealed that the incidence of functional causes resulting in AUB was 65%, whereas only 35% cases were due to organic causes. This is comparable with the findings of Sharma et al. and Dwivedi et al. [8].

The normal cyclical endometrium comprising the proliferative phase endometrium (35%), secretory phase endometrium (18.8%) and menstrual endometrium (3.1%) was seen in 56.9% of total cases. This finding is comparable to studies conducted by Sharma et al. (55.1%) and Vani et al. (56.27%) [5,9]. Behera et al. and Mukhopadhyay et al. have also documented the normal cyclical endometrium as the commonest histopathological pattern observed [7,10]. Among the normal cyclical patterns, the proliferative phase endometrium was documented as the commonest one in most of the studies except for the study done by Sajitha et al. in which secretory phase endometrium was the commonest [6]. The anovulatory cycle is the cause for bleeding in the proliferative phase, and bleeding in the secretory phase is due to ovulatory dysfunction. The finding of normal menstrual phase endometrium accounting for 3.1% of the cases was a unique finding in our study.

In this study, disordered proliferative endometrium was seen in 7.5% of the cases, with the highest incidence in the age group of 18-40 years. This finding was comparable to the study done by Sharma et al. [5]. Doraiswami et al. and Chhatrasal et al. reported a higher incidence of this finding (20.5% and 19.5%, respectively) in their studies [4,11]. The detection of this pattern, which is the earliest stage in the spectrum of proliferative lesions of endometrium including endometrial carcinoma at the other end with intervening stages of hyperplasia, will help to prevent disease progression. Atrophic endometrium was seen in 0.6% of the cases and belonged to the age group of more than 50 years. The incidence of atrophic pattern observed in our study was much lower than that found in other studies. The study by Doraiswami et al. documented an incidence of 2.4% [4]. The highest incidence of this pattern (11%) was seen in the study by Dwivedi et al. and the maximum number of cases with this pattern were in the age group of 50-59 years [8]. Although the exact cause of bleeding in atrophic endometrium is not known, it is postulated to be due to local hemostatic mechanisms. The expanding cystic glands render the overlying thin-walled blood vessels vulnerable to injury.

Among the organic lesions causing AUB, endometrial hyperplasia was found to have the highest incidence constituting 26.3% of the total cases. A total of 20.6% of the cases had hyperplasia without atypia and rest 5.7% were found to have atypical hyperplasia. Endometrial hyperplasia was observed with the highest frequency (23 out of 42) in the age group of 41-50 years. This was the second most common pattern in AUB observed in our study, as in most of the studies. The higher incidence can be attributed to the fact that these patients were not identified at much earlier stages of disordered proliferative endometrium. As endometrial hyperplasia is thought to be a precursor for endometrial carcinoma, the identification of this pattern is very important.

In studies done by Sharma et al., Vani et al., Chhatrasal et al. and Sharma et al., complications of pregnancy were documented as a common cause of abnormal uterine bleeding, especially in the reproductive age group [5,9,11,12]. Hence, while evaluating the endometrial biopsy of patients in this age group presenting with abnormal uterine bleeding, details of the gravindex test for pregnancy should always be checked. In our study, endometrial curettage procedures done for pregnancy-related complications were not included, as in the study done by Samal et al. [13].

Endometrial polyps were found to be the third most common organic lesion causing abnormal uterine bleeding in the present study accounting to 4.4%. The polyps were seen with an equal incidence in reproductive, perimenopausal and post-menopausal age groups. A highest incidence of 11.2% was documented in the study by Doraiswami et al. with most cases in the age group of 41-50 years [4]. The incidence of endometritis found in the present study was 2.5%. One among the four cases was reported as granulomatous endometritis and rest of them were chronic nonspecific endometritis cases. A highest incidence of endometritis was found in the study by Doraiswami et al. in which 1 out of 17 cases was reported as tuberculous endometritis [4].

Endometrial sampling and its histopathological assessment in cases of AUB are mainly done to rule out malignancy. In the present study, the incidence of endometrial carcinoma was 1.2%. One case each was seen in the age group of 41-50 years and post-menopausal age group. A higher incidence of malignancy was reported in studies by Samal et al., Sajitha et al. and Doraisami et al. [4,6,13]. In the study by Doraiswami et al., 18 cases were reported as malignant of which one was malignant mixed Müllerian tumour [4]. Sajitha et al. reported eight cases of malignancy of endometrium of which seven were endometrial carcinoma cases and one was of endometrial stromal sarcoma [6]. Samal et al. reported 13 cases of which 11 were of endometrial adenocarcinoma and 2 were of carcinosarcoma [13]. Although a diagnosis of endometrial malignancy can be made on the endometrial biopsy, a comment on the invasiveness and extent of the tumour cannot be given. Bharani et al. did a study on vanishing carcinomas [14]. An endometrial biopsy-proven carcinoma not showing any tumour in the subsequent hysterectomy specimen is called a vanishing carcinoma. In such cases, prognosis is excellent and adjuvant therapy is not required.

Although endometrial sampling is a simple outpatient procedure to rule out malignancy, scanty endometrial tissue with hemorrhage obscuring the view poses difficulty in providing the histopathological diagnosis.

## Conclusions

AUB is the most common complaint found among the patients in the gynecology outpatient department. The increased awareness and better accessibility to healthcare facilities also contribute to the increase in cases presenting with AUB. AUB requires thorough and prompt evaluation as it can be a clinical manifestation of underlying fatal diseases like endometrial carcinoma. Although at times the interpretation of an endometrial biopsy is quite challenging, a prompt diagnosis made by correlating the clinical history and radiological findings can help in providing the right treatment to the patient. In the present study, we found that women in the perimenopausal age group were the most common to present with AUB. The presenting complaints were variable. The most common histopathological feature was the normal cyclical endometrium including proliferative and secretory phase endometrium. Among the organic lesions, endometrial hyperplasia was the commonest pattern though atypical hyperplasia contributed less. The endometrial polyp was the other common finding. Endometrial carcinomas were found in fewer numbers. Endometrial sampling is an effective diagnostic tool and it needs to be considered in all patients from peri- and post-menopausal age groups with AUB.
